# High-Gain Dual-Polarization Microstrip Antenna Based on Transmission Focusing Metasurface

**DOI:** 10.3390/ma17153730

**Published:** 2024-07-27

**Authors:** Yibo Sun, Bin Cai, Lingling Yang, Ling Wu, Yongzhi Cheng, Hui Luo, Fu Chen, Xiangcheng Li

**Affiliations:** 1 School of Information Science and Engineering, Engineering Research Center for Metallurgical Automation and Detecting Technology Ministry of Education, Wuhan University of Science and Technology, Wuhan 430081, China; sunyb173@163.com (Y.S.); caibin@wust.edu.cn (B.C.); luohui@wust.edu.cn (H.L.); chenfu@wust.edu.cn (F.C.); 2 School of Electronic Engineering, Wuhan Open University, Wuhan 430205, China; yanglingling203@163.com; 3 School of Physics and Electronic Information Engineering, Hubei Engineering University, Xiaogan 432000, China; ruochen143abc@163.com; 4 Key Laboratory of High Temperature Electromagnetic Materials and Structure of MOE, Wuhan University of Science and Technology, Wuhan 430081, China; 5 The State Key Laboratory of Refractories and Metallurgy, Wuhan University of Science and Technology, Wuhan 430081, China

**Keywords:** microstrip antenna, linear polarization, circular polarization, transmission-mode metasurface, high gain

## Abstract

In this paper, a single-feed microstrip antenna (MA) equipped with a transmission-mode focusing metasurface (MS) is proposed to achieve dual-polarization capabilities and superior high-gain radiation performance. The original-feed MA comprises two distinct layers of coaxial-fed tangential patches, enabling it to emit a circular polarization (CP) wave with a gain of 3.5 dBic at 5.6 GHz and linear polarization (LP) radiation with a gain of 4 dBi at 13.7 GHz. To improve the performance of the single-feed MA, a dual-polarization transmission focusing MS is proposed and numerically substantiated. By positioning the originally designed MA at the focal point of the MS, we create a transmission-mode MS antenna system capable of achieving CP and LP radiations with the significantly higher gains of 12.9 dBic and 14.8 dBi at 5.6 GHz and 13.7 GHz, respectively. Measurements conducted on the fabricated dual-polarization focusing MS antenna closely align with the simulation results, validating the effectiveness of our approach. This work underscores the significant potential of dual-polarization high-speed data systems and offers a practical solution for enhancing antenna gains in contemporary wireless communication systems.

## 1. Introduction

In recent years, the advancing maturity of printing and integration processes has propelled microstrip antennas (MAs) to the forefront of research and market applications. Their popularity stems from their low profile, compactness, lightweight design, simplicity in mass production, and conformal characteristics. Nevertheless, a major hurdle in their current development remains, namely, their limited gain performance, which is primarily due to significant losses in matching loads [1,2,3,4]. Furthermore, traditional MAs frequently encounter difficulties in concurrently radiating both linear polarized (LP) and circular polarized (CP) waves, thereby limiting their utilization in multi-functional wireless communications [5,6,7,8]. Consequently, those MAs that employ dual-polarization (LP and CP) models often require a complex feed network with dual (or multiple) ports or the integration of lumped circuits for effective control [9,10,11,12]. For instance, Dasari et al. introduced an MA that integrates positive-intrinsic-negative (PIN) diodes and biased affine function networks to enable three polarization modes: LP, right-hand circular polarization (RCP), and left-hand circular polarization (LCP) [9]. Korošec et al. proposed a design for a reconfigurable MA operating at 0.89 to 1.5 GHz using PIN diodes. This MA can achieve frequency tuning, polarization agility, and phase shifting at 0.89–1.5 GHz [10]. Similarly, Hong et al. introduced a dual-polarization antenna that employs a sophisticated dual-port feed network and switching elements to optimize port isolation [12]. Despite their ability to attain multiple polarization states, these MAs still suffer from challenges related to low gain and radiation efficiency.

Metasurfaces (MSs), being a planar manifestation of two-dimensional (2D) or quasi-2D metamaterials (MMs), have undergone rapid progress across various scientific and engineering disciplines because of their remarkable electromagnetic (EM) characteristics and wide application [13,14,15,16]. An MS offers increased flexibility and simplicity in design and fabrication, driving advancements in a variety of applications, such as absorption [17,18,19,20,21], wavefront manipulation [22,23,24,25,26], polarization conversion [27,28], and numerous others. Specifically, MSs have been extensively utilized in the development of diverse antennas due to their inconspicuous profile, minimal insertion losses, and straightforward implementation [29]. The development of MSs was greatly influenced by antenna theory, and conversely, MSs have played a pivotal role in the antenna domain [30,31,32,33,34,35,36,37]. In recent years, numerous antennas leveraging various types of MSs have been proposed to enhance various performance metrics, including an increased gain [37], broadened bandwidth [38], improved axis ratio (AR) [39], and optimized profile [40]. For example, Han et al. introduced an antenna array equipped with absorbing MSs, achieving a remarkable gain of 15.47 dBi within the operational frequency range [41]. Liang et al. proposed a method for positioning an MA within a multi-layer transmission focusing MS, achieving an average high gain of 16.7 dBi from 9.4 GHz to 10.6 GHz [42]. Hua et al. employed a multi-layer focusing MS to concentrate the feed antenna’s radiation in transmission mode at 15.7 GHz, bidirectional mode at 16.5 GHz, and reflection mode at 17.1 GHz, achieving a gain exceeding 16 dBi [43]. Additionally, Wang et al. introduced a high-gain CP antenna using a focusing MS, achieving a transmission gain of 15.9 dBic at 8.15 GHz and an impressive gain of 19.4 dBic at 14.8 GHz [44]. While the MAs mentioned above that employ MSs demonstrate some level of gain enhancement, they typically function exclusively in either LP or CP mode. To date, there has been no reported instance of dual-polarization antenna systems that incorporate transmission focusing MSs.

In this article, we present a transmission-mode focusing MS antenna that exhibits high-gain and dual-polarization capabilities. Initially, we propose a dual-polarization feed antenna capable of radiating CP waves with a gain of approximately 3.5 dBic at 5.6 GHz and LP waves with a gain of approximately 4 dBi at 13.7 GHz. Subsequently, to enhance the gain performance, we introduce a transmission-mode MS specifically engineered to focus both CP and LP waves at these two specified frequencies, and we verify its effectiveness. The simulation outcomes indicate that the MS exhibits remarkable focusing capabilities for both CP and LP waves at these specific frequencies. Finally, we integrate the transmission-mode focusing MS with the initial feed MA to create a dual-polarization MS antenna system. We fabricated and measured the proposed design, validating the simulation outcomes to reveal a consistency with the simulation outcomes. The gain is significantly improved compared to the initial feed antenna, thereby validating the effectiveness of our proposed MS antenna system.

## 2. The Design of the Feed Antenna and Focusing MS

### 2.1. Dual-Polarization Feed MA

**Figure 1:**
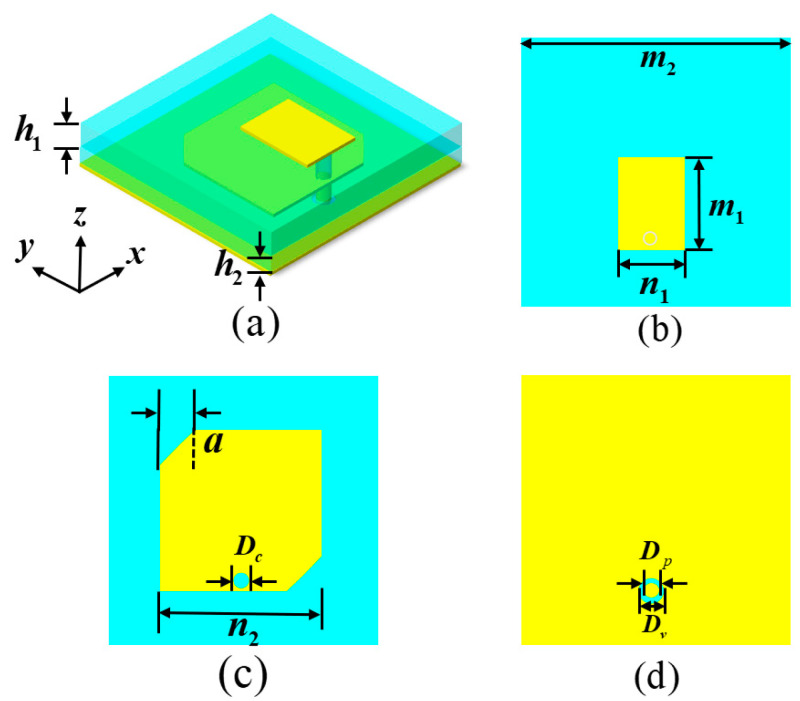
(**a**–**d**) The perspective and front-, middle-, and back-layer views of the designed feed MA.

In this section, we provide an initial design overview and overall performance analysis of the dual-polarization (CP and LP) feed MA. Figure 1a depicts the preliminary design scheme of the dual-polarization feed MA, which comprises three distinct layers of metallic copper, each measuring 0.035 mm in thickness, along with two layers of dielectric substrates (FR-4 board, ε_r_ = 4.4 (1 + I × 0.025)). Illustrated in Figure 1b for the front-layer view of our design, the rectangular metal patch (RMP) structure with a length of m_1_ and a width of n_1_ is directly interfaced with the coaxial probe to produce LP waves at a higher frequency.

**Figure 2:**
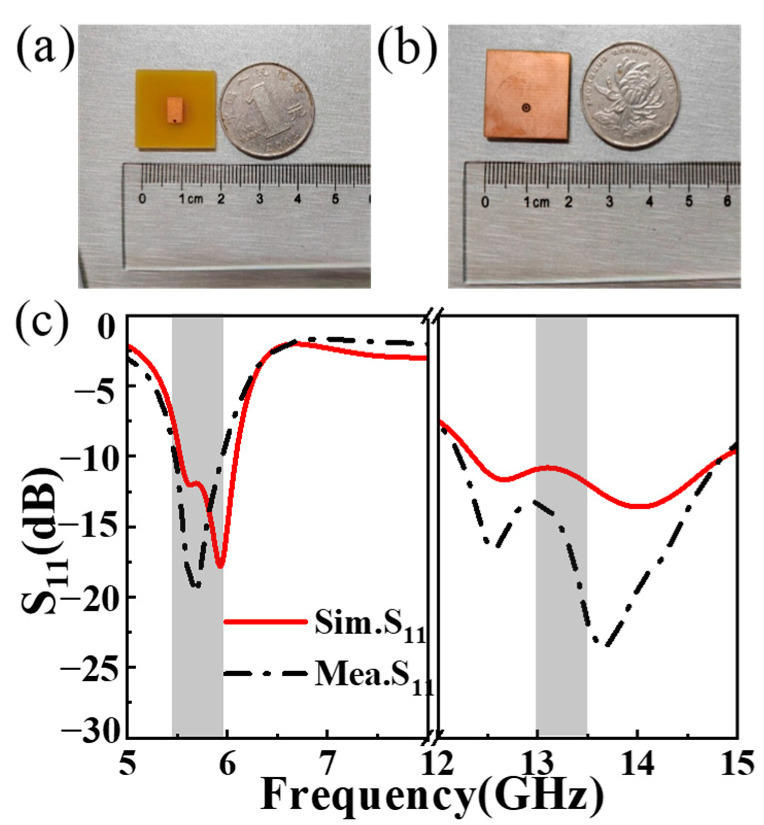
(**a**,**b**) Illustrations of the proposed dual-polarization feed MA sample, and (**c**) the simulated and measured reflection coefficient (S_11_).

In order to realize CP waves at a lower frequency, as shown in Figure 1c, an angular incision is present along the diagonal of the truncated square metallic patch (TSMP) structure, with a side length of *n*_2_ in the middle layer. It can be expected that this designed TSMP structure can radiate the LCP wave [2,3]. It should be noted that if an angular incision is present along another diagonal of the TSMP structure, it has the potential to radiate the RCP wave. This TSMP structure is coupled via the frontal RMP structure and lacks a direct connection to the coaxial probe, and there is a circular slot with a diameter of *D_c_* for passing through it, similar to a previous design [45]. At the same time, the middle and back metal layers can act as the ground layer of the front RMP and middle TSMP structures, respectively, which are used to realize the LP and CP radiation of the feed MA at two different frequency ranges. The operating frequency, designated as *f_r_*, of the feed MA can be predicted utilizing the following formula [3]:

(1) $$f_{r}=\frac{c}{2l_{i}\sqrt{\varepsilon_{eff}}}$$

(2) $$\varepsilon_{eff}=\frac{\varepsilon_{r}+1}{2}$$

where *c* denotes the velocity of light, *l_i_* is side length of the radiant sheet (RMP and TSMP structures), *ε_eff_* signifies the equivalent effective permittivity, while *ε_r_* denotes the relative permittivity of the dielectric substrate. Consequently, the side length of the radiant sheet of the designed feed MA can be estimated based on the fact that the antenna radiates EM waves of the desired frequency range. To substantiate the efficacy of the dual-polarization MA design, a full-wave simulation was executed utilizing the finite-difference time-domain (FDTD) methodology. Throughout the simulation process, open boundary conditions were incorporated surrounding the feed antenna structure, with the corresponding structural geometrical parameters being ascertained through simulation optimization. The definitive geometric parameters for the antenna structure are as follows: *m*_1_ = 6.5 mm, *n*_1_ = 4.6 mm, *m*_2_ = 20 mm, *n*_2_ = 11.3 mm, *a* = 2 mm, *t*_1_ = 1.55 mm, *t*_2_ = 0.95 mm, *D_p_* = 2 mm, *D_c_* = 1 mm, and *D_v_* = 0.6 mm. Ultimately, the designed dual-polarization feed antenna was crafted based on conventional PCB technology, adhering to the aforementioned parameters, and the corresponding samples are depicted in Figure 2a,b. To assess the radiation performance, an advanced vector network analyzer from USA (Agilent E8362B) and an accompanying antenna measuring system were employed [45,46].

Figure 2c provides a depiction of the measured and simulated reflection coefficients (S_11_) of the dual-polarization feed antenna sample, indicating that the S_11_ remains below −10 dB at around 5.4–5.9 GHz and 12.2–14.7 GHz, respectively, revealing the low return loss property of dual bands. In addition, the measured (simulated) S_11_ is about −18.3/−12.1 dB at 5.6 GHz and about −19/−12.01 dB at 13.7 GHz, respectively. It is worth noting that the discrepancies observed between the measured and simulated results are primarily attributed to the following reasons. Firstly, there may be a slight difference in the permittivity of the dielectric substrate. Secondly, there may be minor variations in the thicknesses of the dielectric substrate and metallic structure. Thirdly, the initial microstrip antenna has a finite size in the experimental test environment, whereas this is not the case in simulations.

**Figure 3:**
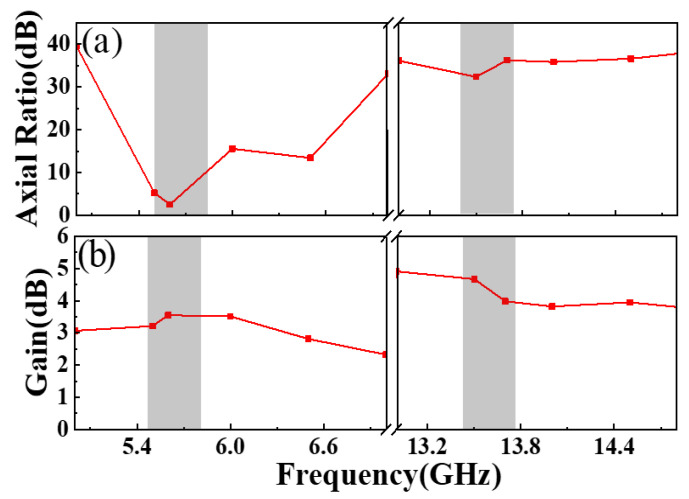
The simulated (**a**) gain and (**b**) axial ratio (AR) of the designed feed antenna.

In order to elucidate the radiation characteristics of the designed feed MA, we present the simulated curves of the axis ratio (AR) and gain, respectively. As depicted in Figure 3a, the AR value is about 2.52 dB at 5.6 GHz and 36.24 dB at 13.7 GHz, respectively, meaning that the designed feed MA can efficiently radiate CP and LP waves at around these frequencies [43,44,45,46,47,48,49]. Obviously, the designed feed MA can achieve a dual-polarization radiation performance within the two desired frequencies. Furthermore, as shown in Figure 3b, the designed feed MA has a gain of 3.5 dBic for the CP wave at 5.6 GHz and a gain of 4 dBi for the LP wave at 13.7 GHz, revealing a relatively lower gain.

**Figure 4:**
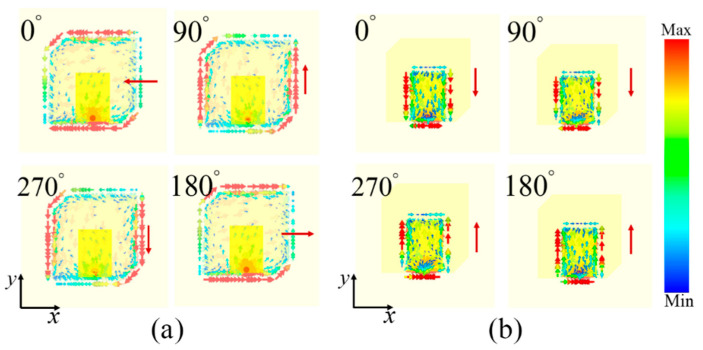
Simulated distributions of surface current with advancing phase in (**a**) the middle-layer TSMP structure at 5.6 GHz and (**b**) the top-layer RMP structure at 13.7 GHz of the proposed dual-polarization feed MA. The red arrow indicates the flow direction of the induced surface current in the radiation elements.

In order to illustrate the operation mechanism of the designed dual-polarization feed antenna, the simulated distributions of the induced surface current with an advancing phase in the middle-layer TSMP structure at 5.6 GHz and the top-layer RMP structure at 13.7 GHz are depicted in Figure 4, respectively. From Figure 4a, at 5.6 GHz, the induced surface current on the TSMP structure of the middle layer rotates clockwise in the forward phase, showing the characteristics of LCP radiation [2,3,47,48,49]. As depicted Figure 4b, at 13.7 GHz, the induced surface current with an advancing phase on the RMP structure follows along the +/−*y*-axis direction, revealing LP radiation. This means that the designed feed MA can effectively radiate the LP wave of the *y*-axis direction at 13.7 GHz and is actually vertical polarization. These distribution features of the surface current with an advancing phase further validate the CP and LP radiation performance of the designed feed antenna across both aforementioned bands [2,3,43,44,45].

**Figure 5:**
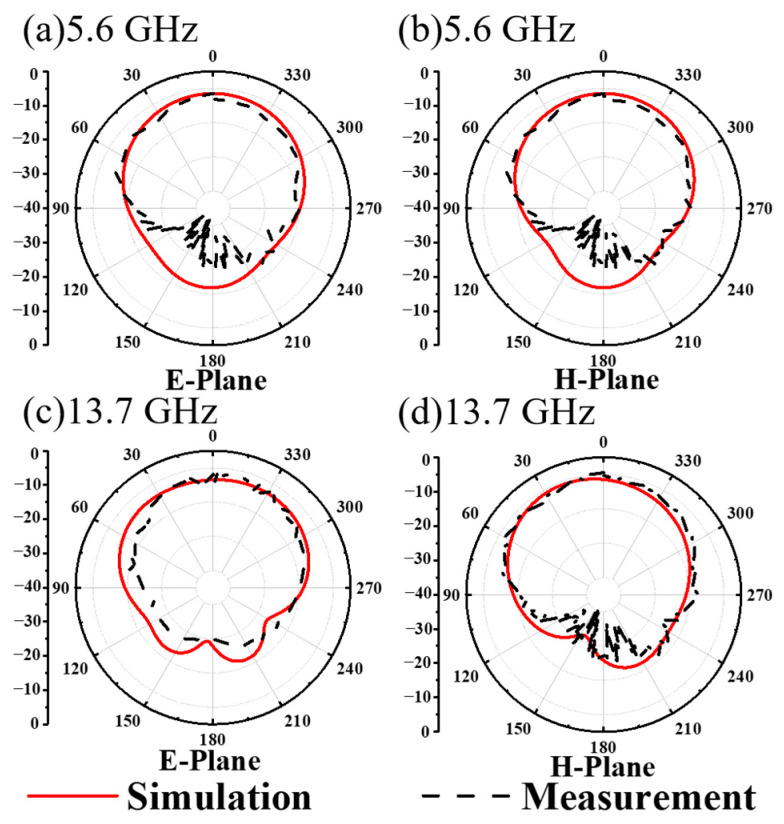
The measured and simulated 2D far radiation patterns of the designed dual-polarization feed antenna in the E- and H-plane at (**a**,**b**) 5.6 GHz and (**c**,**d**) 13.7 GHz.

In order to more fully illustrate the radiation characteristics of the designed feed antenna, as shown in Figure 5, we present the simulated and measured 2D far radiation patterns. It is clear that the measured results are basically consistent with the simulated results, with some minor fluctuations. This may be due to the small size of the initial MA, which is not easy to fix on the rotation axis when measuring the far-field pattern. At 5.6 GHz and 13.7 GHz, the radiation shape of the feed antenna is hemispherical, and the energy is relatively divergent, revealing a lower directionality for both the CP and LP wave radiations.

Obviously, the designed feed antenna can radiate CP and LP waves at two different frequencies, separately. Nevertheless, its practical application is also restricted due to its relatively low gain.

### 2.2. Transmission-Mode Dual-Polarization Focusing MS

Incorporating a focusing MS onto a standard MA is a proven method for optimizing the radiation capabilities of a tailored antenna system, as demonstrated in previous studies [44,45,46,50]. Although antennas using MSs in previous studies show some degree of gain enhancement [35,36,37,38,39,40,41,42,43,44,45,46], they usually only operate in LP or CP mode, or they are enhanced using a reflection focusing MS. So far, there are no reported instances of dual-polarization antenna systems containing a transmission focusing MS. To further enhance these capabilities, we introduce a novel design concept for CP and LP wave transmissive focusing MSs, which aim to augment the radiation performance of the feed MA.

**Figure 6:**
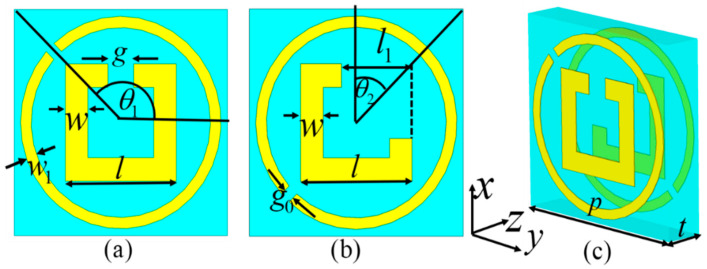
The (**a**–**c**) front, back, and perspective views of the unit cell of the proposed transmission-mode MS.

Figure 6 shows a schematic diagram of the proposed unit cell structure of the transmission-mode MS, composed of two layers of 0.035 mm a copper resonator structure and a single-layer FR-4 dielectric substrate. A single-split-square-ring (SSSR), a diagonal-split-circular-ring (DSCR), and a diagonal-split-square-ring (DSSR) structure are printed on two sides of the dielectric layer of the unit cell. The front layer consists of an external DSCR and an internal SSSR, while the bottom layer consists of an internal DSSR and an external DSCR, in opposition to the top layer. Note that the external DSCRs of front and back layers can form a *C*_2_-rotation symmetric chiral structure and can effectively manipulate CP waves [28,51,52,53,54]. As shown in Figure 6c, the bi-layer reverse DSCR as a chiral structure can act as a CP converter or selector [51,52,53,54], transmuting normal incident LCP/RCP waves into transmitted RCP/LCP waves at 5.6 GHz. The rotation angle of the bi-layer reverse DSCR structure, relative to the *y*-axis direction, is labeled as *θ*_1_, and the opening orientation angle and note angle length of the back DSSR structure with respect to the positive *x*-axis are labeled as *θ*_2_ and *l*_1_, respectively. A combination of the front-layer SSSR structure and the back-layer DSSR structure can function as an LP converter or selector, capable of converting the *y*-/*x*-polarization wave into an *x*-/*y*-polarization wave propagating along the ±*z*-axis direction at 13.7 GHz after transmission. For the MS we designed, the phase of the orthogonal CP wave is primarily controlled by the rotation angle *θ*_1_ of the bi-layer reverse DSCR structure. Therefore, by altering *θ*_1_ of the bi-layer reverse DSCR structure of the MS, an arbitrary phase shift of the orthogonal CP wave, *φ* = 2*θ*_1_, can be realized. Thereby, it can be easily covered completely in the 0~2π phase range. The phase of the transmitted orthogonal LP wave is mainly dictated by the *l*_1_ and *θ*_2_ of the back-layer DSSR structure. Therefore, by adjusting *l*_1_ and *θ*_2_, the phase of the orthogonal LP wave can be easily modulated. Through an adjustment of the dimensions of the SSSR, DSSR, and DSCR structures, crosstalk and interference between orthogonal CP and LP waves in transmission mode can be significantly reduced and eliminated, essentially enabling independent wavefront manipulations in both operation modes.

The highest possible transmission coefficient and polarization conversion efficiency (PCE) within its individual operating bands should be obtained, all while maintaining precise control of the phase corresponding to the emitted orthogonal CP and LP wave. Thus, we elaborated the unit-cell structure to achieve a high PCE and isolation of CP and LP waves. After meticulous optimization, the geometric parameters of the unit cell were obtained: *t* = 2 mm, *p* = 10 mm, *g* = 1.05 mm, *w* = 1.2 mm, *w*_1_ = 0.5 mm, and *l* = 5.2 mm. Adjusting *θ*_1_, *θ*_2_, and *l*_1_ achieves independent control over phases of orthogonal CP and LP waves in transmission mode. A numerical simulation of our proposed MS was performed using CST Microwave Studio. The *x*-/*y*-axis direction of the unit-cell structure was set as the periodic boundary condition, and the *z*-axis direction was set as open (add space). In the simulation, operating frequencies were set at *f*_1_ = 5.6 GHz (*λ*_1_ = 53.5 mm) for the CP wave and *f*_2_ = 13.7 GHz (*λ*_2_ = 21.8 mm) for the LP wave, respectively.

**Figure 7:**
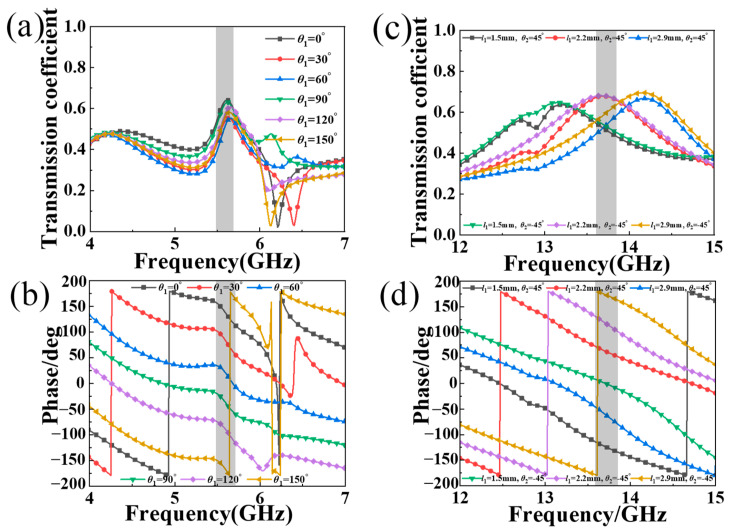
The (**a**,**c**) simulated transmission coefficient and (**b**,**d**) phase of the orthogonal (**a**,**b**) CP wave and (**c**,**d**) LP wave.

As shown in Figure 7, we present the transmission coefficient and phase of the orthogonal CP and LP waves for the proposed MS structure by changing the geometric parameters (*θ*_1_, *θ*_2_, and *l*_1_). As depicted in Figure 7a,c, by adjusting *θ*_1_, the transmission coefficient of the orthogonal CP wave is over 0.5 at around 5.6 GHz, while the corresponding phase can shift linearly, and the adequate full phase shift of 2π can be achieved. From Figure 7b,d, by adjusting *θ*_2_ and *l*_1_, the transmission coefficient of the orthogonal LP wave is also over 0.5 on average at around 13.7 GHz, with an adequate full phase shift of 2π. Thus, the unit-cell structure meets the requirement of establishing CP and LP wave transmission-mode focusing MSs, similar to previous research [26]. Note that the magnitudes of the transmitted CP and LP waves are relatively small (only about 0.6) due to the high dielectric loss of the low-cost FR-4 board. If using the low-loss dielectric substrate board, the transmission efficiency can be enhanced significantly. Based on the aforementioned analysis, the proposed structure is capable of simultaneously regulating CP and LP waves across two distinct frequency bands, thus fulfilling the need for an independent dual-polarization transmission focusing MS [23,24,25,26].

**Figure 8:**
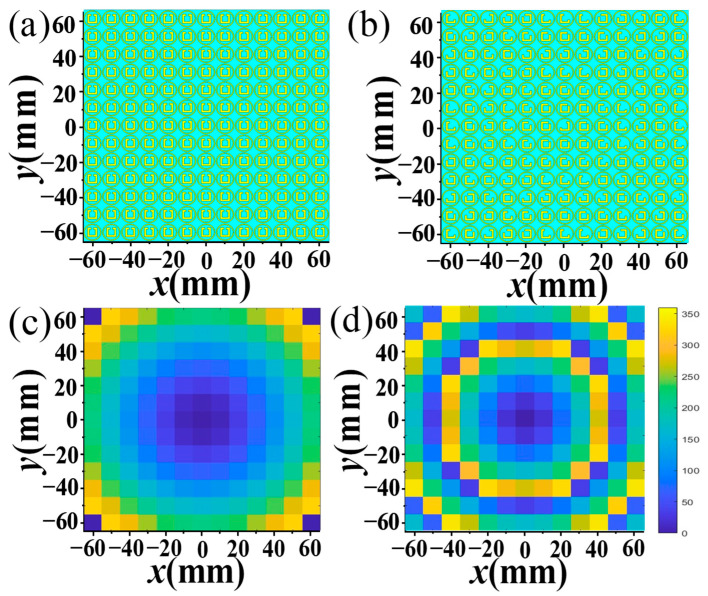
The (**a**) top and bottom (**b**) views of the transmission-mode MS with phase distributions of the (**c**) orthogonal CP wave at 5.6 GHz and (**d**) LP wave at 13.7 GHz.

On the basis of preceding simulation results, we delved into the focusing effect of the CP and LP wave of the designed MS based on the principles of the Pancharatnam–Berry (PB) phase and propagation phase, respectively. By manipulating the phase and transmission coefficient of the orthogonal CP and LP waves, the transmission-mode focusing MS can be constructed. The unit cells, arranged according to the phase distribution formula of focusing MSs, form the transmission-mode focusing MS, as illustrated in Figure 8a,b, which represent the top and bottom layer of the MS, respectively, and the overall size is 130 mm × 130 mm. The formula of the compensation phase distribution of the transmission-mode focusing MS is given by the following [46]:

(3) $$\varphi (x,y)=\frac{2\pi }{\lambda }(\sqrt{x^{2}+y^{2}+F^{2}}-F)$$

where *F* signifies the focal length of the focusing MS; *λ* is the wavelength in free space; and *φ*(*x*, *y*) indicates the phase compensation of the *x*-*y* plane required for different coordinate positions along the *x*- and *y*-axis direction. We chose a focal length *F* of 40 mm to ensure that the overall size of the MS antenna was not too large. Employing Equation (3), a transmission-mode focusing MS was constructed with 13 × 13 unit cells arranged sequentially in the *x*-*y* plane. The simulated phase profiles of transmitted orthogonal CP and LP waves at two distinct frequencies, namely, 5.6 GHz and 13.7 GHz, are illustrated in Figure 8c,d, respectively.

**Figure 9:**
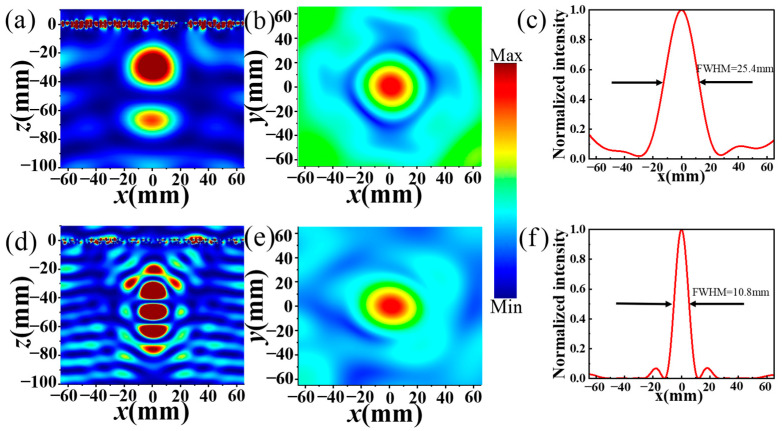
The simulated distributions of (**a**,**b**,**d**,**e**) the electrical field (|*E*|^2^) and the (**c**,**f**) normalized intensity of the transmitted orthogonal (**a**–**c**) CP wave and (**d**–**f**) LP wave in the (**a**,**d**) *x*-*z* and (**b**,**e**) *x*-*y* plane for the CP wave at (**a**–**c**) 5.6 GHz and LP wave at (**d**–**f**) 13.7 GHz.

In order to further verify that the proposed MS has an excellent dual-polarization focusing effect in transmission mode, the simulated electric field in the *x*–*z* and *x*–*y* planes of the transmitted orthogonal CP and LP waves along the *z*-axis and the normalized energy intensity at 5.6 GHz and 13.7 GHz, respectively, are illustrated in Figure 9. As observed in Figure 9a,d, at 5.6 GHz and 13.7 GHz, the focal lengths of the orthogonal CP and LP waves are approximately 38 mm and 39 mm, respectively, approaching the preset value of 40 mm. Furthermore, as revealed by Figure 9b,e, obviously, there are distinct hot spots located at the center of the *xoy* plane (focal plane) for the transmitted orthogonal CP and LP waves at frequencies of 5.6 GHz and 13.7 GHz, respectively. This indicates that when the incident CP and LP waves pass through the designed MS, they are effectively concentrated and focused onto a single point. Figure 9c,f reveal that the calculated full width half maximums (FWHMs) of the electric intensity along the *x*-axis direction of the transmitted orthogonal CP and LP waves are 25.4 mm and 10.8 mm, respectively. These results underscore the outstanding dual-polarization focusing effect of the designed transmission-mode MS.

## 3. Dual-Polarization Transmission-Mode Focusing MS Antenna

**Figure 10:**
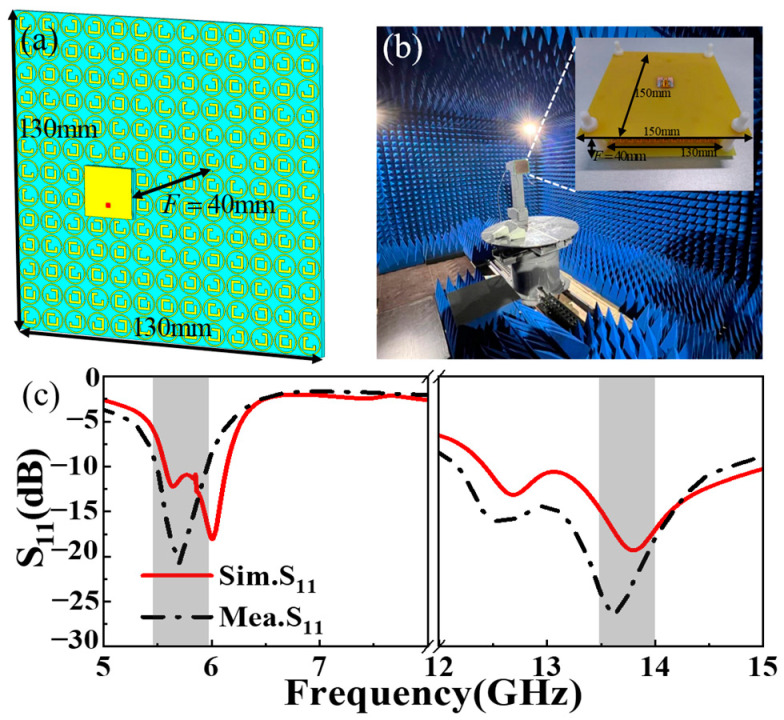
The (**a**) configuration of the dual-polarization MS antenna system, the (**b**) test environment and corresponding sample, and a (**c**) comparison of the measured and simulated reflection coefficient (S_11_).

Adding and placing a dual-polarization feed antenna at the focal point of an MS is an effective way to improve performance. As discussed in the previous section, the transmission-mode MS has the ability to converge a plane wave towards a focal point. Consistent with the principle of the reversibility of EM waves, the MS can subsequently transform the spherical wave emanating from this focal point back into a plane wave. This section aims to illustrate the functionality and performance of the MS antenna based on a combination of the above-designed dual-polarization transmissive focusing MS and MA. As illustrated in Figure 10a, the proposed dual-polarization feed MA is placed along the *+z*-axis at a distance of 40 mm from the center of the transmission-mode MS.

In order to better fix the feed antenna and the transmission-mode MS, both the initial feed MA dielectric layer and the MS layer are uniformly enlarged during processing and are fixed with screws, and the final fabricated MS antenna system and test environment are depicted in Figure 10b. The reflection coefficient (S_11_) and radiation performance of the designed MS antenna were measured utilizing an Agilent E8362B vector network analyzer and MA measurement system in the anechoic chamber.

Figure 10c illustrates the simulated and measured S_11_ of the proposed MS antenna. The measured S_11_ is consistent with the simulated result, exhibiting minor discrepancies primarily attributable to manufacturing imperfections, fluctuations in welding procedures, and environmental disturbances during the testing process. The performance of the designed MS antenna remained largely unaffected across resonance frequencies and within adjacent frequencies. The value of the simulated (measured) S_11_ is −11.4/−12 dB at 5.6 GHz and −18.6/−23.2 dB at 13.7 GHz, respectively. Note that the S_11_ of the MS antenna changes a little compared to the initial MA, which is due to the parasitic coupling effect that exists between the MS and the initial MA. The aforementioned results affirm that the designed MS antenna system can be effectively tailored to accommodate the two desired frequency bands.

**Figure 11:**
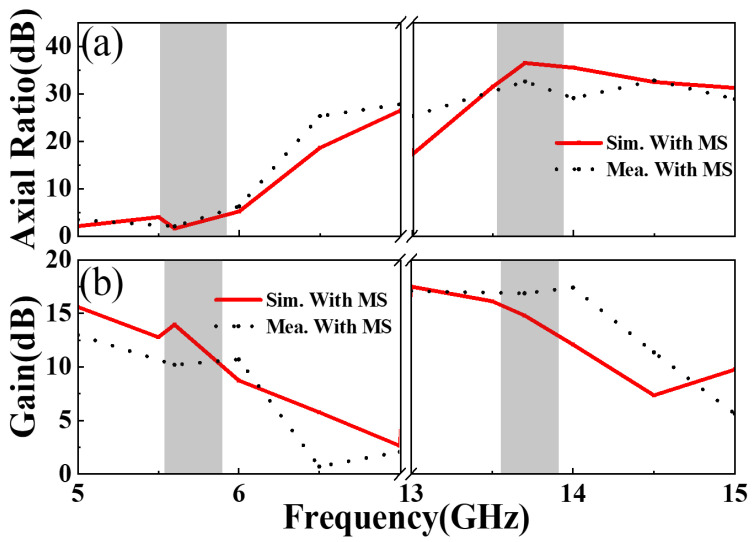
The measured and simulated (**a**) AR and (**b**) gain of the proposed dual-polarization MS antenna.

From Figure 11a, both the measured and simulated ARs are less than 3 dB at around 5.6 GHz, while one is over 30 dB at around 13.7 GHz. The above results prove that the designed MS antenna maintains the CP and LP characteristics of the MA at 5.6 GHz and 13.7 GHz, respectively. It also means that the polarization characteristics of the MS antenna system are not significantly changed compared with the previously designed MA, and CP and LP waves can be effectively radiated above two frequencies. It is worth noting that at this time, the MS antenna system radiates RCP waves at 5.6 GHz, which is due to the cross-polarization conversion of the EM waves emitted by the feed MA as they pass through the focusing MS. As the dual-polarization transmission-mode focusing MS is capable of transforming the spherical waves emitted by the initial MA at the focal point into plane waves [38,43,44,45,46,48,49,50], the gain of the MS antenna exhibits significant improvements over the initial MA across both operating bands. Figure 11b illustrates that the simulated (measured) gain of the MS antenna is up to 12.9(10.2) dBic at 5.6 GHz and 14.8(16.8) dBi at 13.7 GHz, respectively, representing a significant improvement compared to the previously designed MA. This means that the gain enhancement of the MS antenna is over 6 dBic at 5.6 GHz for CP radiation, and the gain is boosted by over 8 dBi at 13.7 GHz for LP radiation after loading the transmissive focusing MS. The implementation of the focusing MS demonstrates a notable enhancement in the gain amplification of the proposed MS antenna system.

**Figure 12:**
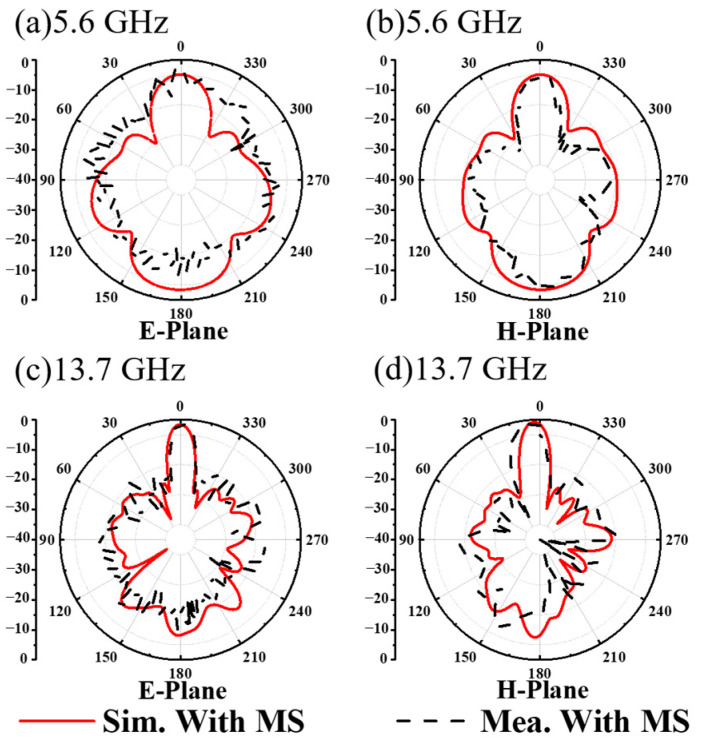
The measured and simulated 2D far-field radiation patterns of the dual-polarization MS antenna in the E plane and H plane at (**a**,**b**) 5.6 GHz and (**c**,**d**) 13.7 GHz.

As shown in Figure 12, the simulated and measured 2D far-field radiation patterns are presented at 5.6 GHz and 13.7 GHz, respectively. This is further evidence that the proposed MA has directional radiation properties that are specific to both CP and LP waves. Note that the measured far-field radiation pattern is slightly impacted by interference from clutter in the test environment and noise from the test system, resulting in a jittery, sawtooth-like effect. Although this introduces some deviation from the simulated results, the overall trend remains intact. The above-simulated and -measured dates show that the MS antenna has the ability of radiating CP and LP waves at two frequencies, respectively, to achieve high-gain radiation. As demonstrated, the proposed MS antenna displays exceptional dual-polarization gain amplification across two frequencies. The experimental results confirm that the focusing MS effectively concentrates microwave energy into a focused beam in transmission mode, achieving the desired effect at around 5.6 GHz for CP waves and at around 13.7 GHz for LP waves. This innovative approach offers a promising solution for dual-polarization radiation in communication systems.

In order to better understand the advantages of the proposed MS antenna, in Table 1, it is compared with other existing antennas loaded with focusing MSs, accordingly. In previous reported works [36,39,43,44,45,46,50,55,56,57,58,59], the MS antenna only realizes single polarization focusing or works in reflection mode. Due to the reflection-mode focusing MS antenna, the EM waves are blocked by the feed, but the transmission-mode MS does not have this defect, which is more advantageous. Undeniably, our proposed transmission-mode focused MS antenna design significantly outperforms its competitors in terms of dual-polarization radiation and high-gain implementation, offering superior performance overall.

## 4. Conclusions

In conclusion, a feed MA loaded with a dual-polarization transmission-mode focusing MS is proposed and demonstrated. The initial feed MA comprises two distinct layers of coaxial-fed tangential patches, and the corresponding gains are 3.5 dBic and 4 dBi at 5.6 GHz and 13.7 GHz, respectively. To optimize the radiation performance of the initial feed MA, a dual-polarization transmission-mode MS was introduced. The metal SSSR, DSCR, and DSSR structures are printed on two sides of the dielectric layer of the unit cell to construct the designed MS based on the principles of the PB phase and propagation phase, respectively, which are capable of focusing LP and CP waves above two distinct frequencies. After loading the designed dual-polarization transmission focusing MS, the MS antenna can radiate 12.9 dBic for the CP wave at 5.6 GHz and 14.8 dBi for the LP wave at 13.7 GHz. Through sample processing and measuring, the results correspond closely with the simulation ones. The proposed MS antenna exhibits superior dual-polarization and high-gain radiation characteristics, providing an effective solution for dual-polarization radiation and high-speed data transmission in communication systems.

## Figures and Tables

**Table 1:** A performance comparison of the designed MS antenna to previous ones.

Ref.	Polarization	Frequency (GHz)	Peak Gain	With Diodes	Gain-Enhanced Method
[35]	LP	22.3–40	9 dBi (LP)	No	Reflective focusing MS
[38]	LP	5.7	9.3 dBi (LP)	No	Huygens MS
[42]	CP	9.4–10.6	16.7 dBi (LP)	No	Reflective focusing MS
[43]	LP	15.7/16.5/17.1	20.4 dBi (LP)	No	Bidirectional focusing MS
[44]	CP	8.15/14.8	19.4 dBic (CP)	No	Full-space focusing MS
[50]	CP	5	7.6 dBic (CP)	Yes	Reflective focusing MS
This work	CP/LP	5.6/13.7	12.9 dBi (LP)/14.8 dBic (CP)	No	Transmission focusing MS

## Data Availability

The raw data supporting the conclusions of this article will be made available by the authors on request.

## References

[1] Li W., Liu S., Deng J., Hu Z. (2020). A compact SIW monopulse antenna array based on microstrip feed. IEEE Antennas Wireless Propag. Lett..

[2] Chen C. (2023). An Ultraminiature Circularly Polarized Microstrip Patch Antenna with MIM Capacitors. IEEE Antennas Wireless Propag. Lett..

[3] Hussain N., Jeong M.J., Abbas A., Kim T.J., Kim N. (2020). A metasurface-based low-profile wideband circularly polarized patch Antenna for 5G millimeter-wave systems. IEEE Access.

[4] Bancroft R. (2021). Microstrip antenna efficiency and surface wave loss. IEEE Trans. Antennas Propag..

[5] Huang D., Xu G., Wu J., Wang W., Yang L., Huang Z.X., Wu X.L., Yin W.Y. (2022). A microstrip dual-split-ring antenna array for 5G millimeter-wave dual-band applications. IEEE Antennas Wireless Propag. Lett..

[6] Pandey B.K., Chakrabarty S.B., Jyoti R. (2018). Common aperture dual-band dual-polarized planar microstrip antenna. IETE J. Res..

[7] Roy C., Khan T. (2019). Single-feed dual-polarized high gain microstrip antenna. Wireless Pers. Commun..

[8] Ding X.H., Yang W.W., Tang H., Guo L., Chen J.X. (2022). A dual-band shared-aperture antenna for microwave and millimeter-wave applications in 5G wireless communication. IEEE Trans. Antennas Propag..

[9] Dasari R.K., Pandharipande V.M., Koul S.K. (2014). A new microstrip patch antenna with triple-polarization diversity. Microw. Opt. Techn. Let..

[10] Korošec T., Ritoša P., Vidmar M. (2006). Varactor-tuned microstrip-patch antenna with frequency and polarization agility. Electron. Lett..

[11] Cao W.Q., Wang Q.Q., Zhang B.N., Hong W. (2017). Capacitive probe-fed compact dual-band dual-mode dual-polarisation microstrip antenna with broadened bandwidth. IET Microw. Antenna. Propag..

[12] Hong Y.P., Kim J.M., Jeong S.C., Kim D.H., Yook J.G. (2005). Low profile S-band dual-polarized antenna for SDARS application. European Conference on Wireless Technology, 2005.

[13] Zahra S., Ma L., Wang W., Li J., Chen D., Liu Y., Wen G. (2021). Electromagnetic metasurfaces and reconfigurable metasurfaces: A review. Front. Phys..

[14] Deng M., Cotrufo M., Wang J., Dong J., Ruan Z., Alù A., Chen L. (2024). Broadband angular spectrum differentiation using dielectric metasurfaces. Nat. Commun..

[15] Ju Z.Z., Wen J., Shi L.N., Yu B.B., Deng M., Zhang D.W., Hao W.M., Wang J., Chen S.Q.., Chen L. (2021). Ultra-Broadband High-Efficiency Airy Optical Beams Generated with All-Silicon Metasurfaces. Adv. Opt. Mater..

[16] Huang Z.R., Zheng Y.Q., Li J.H., Cheng Y.Z., Wang J., Zhou Z.K., Chen L. (2023). High-Resolution Metalens Imaging Polarimetry. Nano Lett..

[17] Wang Q., Cheng Y.Z. (2020). Compact and low-frequency broadband microwave metamaterial absorber based on meander wire structure loaded resistors. AEU-Int. J. Electron. Commun..

[18] Li Z.R., Cheng Y.Z., Luo H., Chen F., Li X. (2022). Dual-band tunable terahertz perfect absorber based on all-dielectric InSb resonator structure for sensing application. J. Alloys Compd..

[19] Ding Z.P., Su W., Luo Y., Ye L., Li W., Zhou Y., Tang B., Yao H. (2023). Artificial neural network-based inverse design of metasurface absorber with tunable absorption window. Mater. Des..

[20] Cai B., Wu L., Zhu X., Cheng Z.Z., Cheng Y.Z. (2024). Ultra-broadband and wide-angle plasmonic light absorber based on all-dielectric gallium arsenide (GaAs) metasurface in visible and near-infrared region. Results Phys..

[21] Wu L., Yang L.L., Zhu X.W., Cai B., Cheng Y.Z. (2024). Ultra-broadband and wide-angle plasmonic absorber based on all-dielectric gallium arsenide pyramid nanostructure for full solar radiation spectrum range. Int. J. Therm. Sci..

[22] Gorkunov M.V., Kasyanova I.V., Artemov V.V., Ezhov A.A., Mamonova A.V., Simdyankin I.V., Palto S.P. (2020). Superperiodic liquid-crystal metasurfaces for electrically controlled anomalous refraction. ACS Photonics.

[23] He Y.Q., Cai B., Wu L.L., Chen L., Cheng Y.Z., Chen F., Luo H., Li X.C. (2024). Tunable VO2 metasurface for reflective terahertz linear and circular polarization wavefront manipulation at two frequencies independently. Phys. B.

[24] Yin X., Zhu H., Guo H., Deng M., Xu T., Gong Z., Li X., Zhang Z.H., Wu C., Li H. (2019). Hyperbolic Metamaterial Devices for Wavefront Manipulation. Laser Photonics Rev..

[25] Yang D.R., Cheng Y.Z., Luo H., Chen F., Wu L. (2023). Ultra-thin and ultra-broadband terahertz single-layer metasurface based on double-arrow-shaped resonator structure for full-space wavefront manipulation. Adv. Theor. Simul..

[26] Li J., Cheng Y.Z., Li X.C. (2022). Terahertz transmission-type metasurface for the linear and circular polarization wavefront manipulation. Adv. Theory Simul..

[27] Wu T., Chen J., Wang M.J. (2020). Multi-state circularly polarized antenna based on the polarization conversion metasurface with gain enhancement. IEEE Access.

[28] Zhao J.C., Li N., Cheng Y.Z. (2023). Ultrabroadband chiral metasurface for linear polarization conversion and asymmetric transmission based on enhanced interference theory. Chin. Opt. Lett..

[29] Wang J., Li Y., Jiang Z.H., Shi T., Tang M.C., Zhou Z., Chen Z.N., Qiu C.W. (2020). Metantenna: When metasurface meets antenna again. IEEE Trans. Antennas Propag..

[30] Boyarsky M., Sleasman T., Imani M.F., Gollub J.N., Smith D.R. (2021). Electronically steered metasurface antenna. Sci. Rep..

[31] Faenzi M., González-Ovejero D., Maci S. (2020). Flat gain broadband metasurface antennas. IEEE Trans. Antennas Propag..

[32] Bodehou M., Monnoyer G., Drouguet M., Khalifeh K.A., Vandendorpe L., Craeye C. (2022). Metasurface Antennas for FMCW Radar. IEEE Antennas Wireless Propag. Lett..

[33] Benini A., Barrera A.M., Martini E., Toccafondi A., Maci S. (2023). Self-complementary hyperbolic metasurface antennas. IEEE Trans. Antennas Propag..

[34] Zhu B., Yang D., Pan J., Chen Y., Liu S. (2023). A Low-Profile Metasurface-Inspired Antenna With Tilted Beam Radiation. IEEE Antennas Wireless Propag. Lett..

[35] Tahir M.U., Rafique U., Ahmed M.M., Abbas S.M., Iqbal S., Wong S.W. (2023). High gain metasurface integrated millimeter-wave planar antenna. Int. J. Microw. Wirel. Trans..

[36] Lian J.W., Ding D., Chen R. (2022). Wideband millimeter-wave substrate-integrated waveguide-fed metasurface antenna. IEEE Trans. Antennas Propag..

[37] Feng M., Li Y., Wang J., Zheng Q., Sui S., Wang C., Chen H., Ma H., Qu S., Zhang J. (2018). Ultra-wideband and high-efficiency transparent coding metasurface. Appl. Phys. A.

[38] Chen K., Yang Z., Feng Y., Zhu B., Zhao J., Jiang T. (2015). Improving microwave antenna gain and bandwidth with phase compensation metasurface. AIP Adv..

[39] Chen Z.N., Qing X. (2016). Bandwidth enhancement of a single-feed circularly polarized antenna using a metasurface. IEEE Antenn Propag. Mag..

[40] Epstein A., Wong J.P., Eleftheriades G.V. (2016). Cavity-excited Huygens’ metasurface antennas for near-unity aperture illumination efficiency from arbitrarily large apertures. Nat. Commun..

[41] Han Y., Zhu L., Bo Y., Che W., Li B. (2019). Novel low-RCS circularly polarized antenna arrays via frequency-selective absorber. IEEE Trans. Antennas Propag..

[42] Liang J.J., Huang G.L., Zhao J.N., Gao Z.J., Yuan T. (2019). Wideband phase-gradient metasurface antenna with focused beams. IEEE Access.

[43] Hua L.N., Cheng H.Y., Wang Y.C., Yang H., Liu Y., Yang Y., Li S. (2021). Bidirectional radiation high-gain antenna based on phase gradient metasurface. Appl. Phys. B.

[44] Wang J.X., Cheng Y.Z., Luo H., Chen F., Wu L. (2022). High-gain bidirectional radiative circularly polarized antenna based on focusing metasurface. AEU-Int. J. Electron. Commun..

[45] Wang J.X., Zhao J.C., Cheng Y.Z., Luo H., Chen F. (2022). Dual-band high-gain microstrip antenna with a reflective focusing metasurface for linear and circular polarizations. AEU-Int. J. Electron. Commun..

[46] Zhou E.Y., Cheng Y.Z., Luo H., Chen F. (2021). Wideband and high-gain patch antenna with reflective focusing metasurface. AEU-Int. J. Electron. Commun..

[47] Saini R.K. (2022). A Broadband Dual Circularly Polarized Compact Printed Monopole Antenna. Wireless Pers. Commun..

[48] Xie P., Wang G. (2023). Gain enhancement of circularly polarized Fabry-Perot resonator antenna using simple superstrate. AEU-Int. J. Electron. Commun..

[49] Liu S.F., Xue Y., Li K.Z. (2023). A dual-slot Vivaldi antenna with high gain and circular polarization. Chin. J. Radiol..

[50] Kumar M.V., Sharma D. (2023). Design of High Gain Metasurface Antenna Using Hybrid African Vulture’s Optimization and Capuchin Search Algorithm for RF Energy Harvestin. Wireless Pers. Commun..

[51] Li A., Chen W., Wei H., Lu G., Alù A., Qiu C.W., Chen L. (2022). Riemann-encircling exceptional points for efficient asymmetric polarization-locked devices. Phys. Rev. Lett..

[52] Shu X., Zhong Q., Hong K., You O., Wang J., Hu G., Alù A., Zhang S., Christodoulides D.N., Chen L. (2024). Chiral transmission by an open evolution trajectory in a non-Hermitian system. Light-Sci. Appl..

[53] Shu X., Li A., Hu G., Wang J., Alù A., Chen L. (2022). Fast encirclement of an exceptional point for highly efficient and compact chiral mode converters. Nat. Commun..

[54] Ferreira-Gomes B., Oliveira J.O.N., Mejía-Salazar J.R. (2021). Chiral dielectric metasurfaces for highly integrated, broadband circularly polarized antenna. Sensors.

[55] Li X., Wang Y., Fan J., He J., Huang X. (2022). Ultra-thin/wide-band polarization conversion metasurface and its applications in anomalous reflection and RCS reduction. Appl. Sci..

[56] Li B., Chen Y., Yu S., Zhou X., Wu Q., Wang J., Li Y., Li F. (2023). Ultra-thin broadband circular polarization conversion metasurface for full-space wavefront manipulation application. IEEE Photonics J..

[57] El-Hakim H., Mohamed H.A. (2023). Synthesis of a Multiband Microstrip Patch Antenna for 5G Wireless Communications. J. Infrared Millim. Terahertz Waves.

[58] Chen Q., Li J.Y., Yang G., Cao B., Zhang Z. (2019). A polarization-reconfigurable high-gain microstrip antenna. IEEE Trans. Antennas Propag..

[59] Zhou E.Y., Cheng Y.Z., Chen F., Luo H., Li X. (2022). Low-profile high-gain wideband multi-resonance microstrip-fed slot antenna with anisotropic metasurface. Prog. Electromagn. Res..

